# Evaluating the effectiveness of abbreviated breast MRI (abMRI) interpretation training for mammogram readers: a multi-centre study assessing diagnostic performance, using an enriched dataset

**DOI:** 10.1186/s13058-022-01549-5

**Published:** 2022-07-30

**Authors:** Lyn I. Jones, Andrea Marshall, Premkumar Elangovan, Rebecca Geach, Sadie McKeown-Keegan, Sarah Vinnicombe, Sam A. Harding, Sian Taylor-Phillips, Mark Halling-Brown, Christopher Foy, Elizabeth O’Flynn, Hesam Ghiasvand, Claire Hulme, Janet A. Dunn, the FAST MRI Study Group, Christiane Kuhl, Jennifer Wookey, Janice Rose, Victoria Taylor, John Gifford, Rosie Gray, Thomas William-Jones, Karen Litton, Simon Lloyd, Jim Steel, Elisabeth Kutt, Alexandra Valencia, Alice Pocklington, Anjum Mahatma, Helen Massey, Gillian Clark, Clare McLachlan, Gemini Beckett, Clare Alison, Miklos Barta, Claudia Betancourt, Julie Bramwell, Nichola Bright, Helen Burt, Louise Cann, Jane Ceney, Eleanor Cornford, Diana Dalgliesh, Sarah Doyle, Sarah Fearn, Dagmar Godden, Zoe Goldthorpe, Lucinda Hobson, Paul Hynam, Emma Jackson, Margaret Jenkin, Beckie Kingsnorth, Katherine Klimczak, Alice Moody, Sarah Perrin, Alison Peters, Elizabeth Preston, Anne Ratsey, Richard Sidebottom, Lesley Stephenson, Michelle Taylor, Erika Toth, Frances Vincent, Sharon Watkin, Sue Widdison, Jennifer Williams, Karen Wilmot, Sravya Singamaneni, Zsolt Friedrich, Joanne Robson, Elizabeth Cullimore, Anna Mankelow

**Affiliations:** 1 North Bristol NHS Trust, Southmead Hospital, Southmead Road, Westbury on Trym, Bristol, BS10 5NB UK; 2 Warwick Clinical Trials Unit, University of Warwick, Coventry, CV4 7AL UK; 3 Scientific Computing, Royal Surrey County Hospital NHS Foundation Trust, Guildford, GU2 7XX UK; 4 Gloucestershire Hospitals NHS Foundation Trust, Cheltenham, GL53 7AS UK; 5 Research Design Service South West Gloucester Office, National Institute for Health Research (NIHR) Leadon House, Gloucestershire Royal Hospital, Gloucester, GL1 3NN UK; 6 St George’s University Hospitals Foundation Trust, London, SW17 0QT UK; 7 Institute of Health Research, University of Exeter Medical School, Exeter, EX1 2LU UK

**Keywords:** Breast cancer, Abbreviated breast MRI, FAST MRI, Education, Training, Diagnostic accuracy

## Abstract

**Background:**

Abbreviated breast MRI (abMRI) is being introduced in breast screening trials and clinical practice, particularly for women with dense breasts. Upscaling abMRI provision requires the workforce of mammogram readers to learn to effectively interpret abMRI.

The purpose of this study was to examine the diagnostic accuracy of mammogram readers to interpret abMRI after a single day of standardised small-group training and to compare diagnostic performance of mammogram readers experienced in full-protocol breast MRI (fpMRI) interpretation (Group 1) with that of those without fpMRI interpretation experience (Group 2).

**Methods:**

Mammogram readers were recruited from six NHS Breast Screening Programme sites. Small-group hands-on workstation training was provided, with subsequent prospective, independent, blinded interpretation of an enriched dataset with known outcome. A simplified form of abMRI (first post-contrast subtracted images (FAST MRI), displayed as maximum-intensity projection (MIP) and subtracted slice stack) was used. Per-breast and per-lesion diagnostic accuracy analysis was undertaken, with comparison across groups, and double-reading simulation of a consecutive screening subset.

**Results:**

37 readers (Group 1: 17, Group 2: 20) completed the reading task of 125 scans (250 breasts) (total = 9250 reads). Overall sensitivity was 86% (95% confidence interval (CI) 84–87%; 1776/2072) and specificity 86% (95%CI 85–86%; 6140/7178). Group 1 showed significantly higher sensitivity (843/952; 89%; 95%CI 86–91%) and higher specificity (2957/3298; 90%; 95%CI 89–91%) than Group 2 (sensitivity = 83%; 95%CI 81–85% (933/1120) *p* < 0.0001; specificity = 82%; 95%CI 81–83% (3183/3880) *p* < 0.0001). Inter-reader agreement was higher for Group 1 (kappa = 0.73; 95%CI 0.68–0.79) than for Group 2 (kappa = 0.51; 95%CI 0.45–0.56). Specificity improved for Group 2, from the first 55 cases (81%) to the remaining 70 (83%) (*p* = 0.02) but not for Group 1 (90–89% *p* = 0.44), whereas sensitivity remained consistent for both Group 1 (88–89%) and Group 2 (83–84%).

**Conclusions:**

Single-day abMRI interpretation training for mammogram readers achieved an overall diagnostic performance within benchmarks published for fpMRI but was insufficient for diagnostic accuracy of mammogram readers new to breast MRI to match that of experienced fpMRI readers. Novice MRI reader performance improved during the reading task, suggesting that additional training could further narrow this performance gap.

**Supplementary Information:**

The online version contains supplementary material available at 10.1186/s13058-022-01549-5.

## Background

Breast cancer screening in most western countries predominantly uses digital mammography technology, with full-protocol breast MRI (fpMRI) used for some high-risk groups. Randomised controlled screening trials of abbreviated breast MRI (abMRI) from the USA [1] and of fpMRI from Europe [2, 3] have demonstrated the potential use of abMRI for screening a wider group of women than those currently screened with fpMRI. In response, abMRI has been introduced into clinical practice to screen women with either mammographically dense breasts or other reasons for being above population risk of breast cancer [4–10].

The diagnostic accuracy of abMRI is similar to that of fpMRI when reported by professionals expert in fpMRI interpretation [11–19], whilst shorter acquisition (3–13 min abMRI vs. 15-32 fpMRI) and reading times for abMRI (0.6–3 min vs. 3–7) promise potential cost savings in comparison with fpMRI [11–14, 16, 18, 20, 21]. However, important unknowns include the feasibility of upscaling abMRI capacity [20, 22]. Assessing the capacity of the workforce to interpret the additional abMRI scans is an essential part of feasibility assessment.

Learning fpMRI interpretation is an apprenticeship-style process, taking several years to obtain accredited skills. The American College of Radiologists (ACR), European Society of Breast Imaging (EUSOBI) and the UK’s National Breast Imaging Academy consider 100 documented/logged fpMRI interpretations over 1–2 years’ accredited learning to be sufficient to demonstrate proficiency in fpMRI reporting [23–25]. Internationally, the professional group best placed to augment numbers of existing fpMRI interpreters to read screening abMRI may be readers of screening mammograms, for whom additional training, accreditation and quality assurance would be required [10, 26].

A previous single-centre study, of 8 readers from the UK National Health Service Breast Screening Programme (NHSBSP), suggested that NHSBSP mammogram readers could be effectively trained to interpret abMRI with a single day’s one-to-one training [21, 27]. Other international publications describe specific abMRI interpretation training for radiologists reading abMRI [1, 28, 29], but there is little published evidence evaluating abMRI interpretation training. To address this knowledge gap with formal evaluation of abMRI small-group training, our previous study’s one-to-one training [21] was adapted to create an electronic training package, delivered as single-day, small-group, in-person, hands-on workstation training. We present the results of a multi-centre study designed to evaluate the impact of the training on mammogram readers and to compare diagnostic performance of mammogram readers experienced in fpMRI interpretation with that of those without fpMRI interpretation experience.

## Methods

This study was reviewed and approved by the London-Bromley Research Ethics Committee and by the Health Research Authority (England and Wales) (REC: 19/LO/1473 IRAS:258203), and prospectively registered (ISRCTN:16624917), and all participants gave written informed consent.

### Aims

To examine the diagnostic accuracy of mammogram readers to interpret abMRI after a single day of standardised small-group training and to compare diagnostic performance of mammogram readers experienced in fpMRI interpretation with that of those without fpMRI interpretation experience.

### Study design

Prospective, blinded interpretation of an enriched dataset by multiple readers.

### Participants and setting

NHSBSP multi-professional mammogram readers, fully qualified to interpret mammograms [30], at 6 sites (NHSBSP screening units) within the South-West Region of England were invited (September–December 2019) and classified as Group 1 if they also interpreted fpMRI in their normal clinical practice, and Group 2 if not. Participants attended a single day of standardised training (October 2019–January 2020) and then interpreted a test set of abMRI scans (January–July 2020).

### Test set

The test set comprised 125 abMRIs with known outcome acquired as fpMRI during 2015: 72 consecutive high-risk screening scans [31] (including two with unilateral cancer) enriched with 53 additional cancer cases from consecutive fpMRI scans acquired at cancer diagnosis (reported as unifocal invasive cancer ≤ 25mm or ductal carcinoma in situ (DCIS) of any size). Of the two cancers within the high-risk screening series of 72 scans, one was detected from the 2015 fpMRI (screen-detected) but the other was not recognised in 2015 (interval cancer). All cancers had histological confirmation, and non-cancer scans had 2-year minimum follow-up. Test set composition, imaging and display protocol were previously described [21] (abMRI specification and test set composition reproduced in Additional file 1: Appendix 1). Of 125 abMRIs in the dataset, 54 had biopsy-confirmed unilateral cancer and one bilateral (56 breasts with cancer) and 2 women had two separate tumours identified in the same breast, giving a total of 58 cancers reported in the ground truth, 56 invasive and 2 ductal carcinoma in situ (DCIS) [21]. The mean, median and range of invasive cancer size was 15.7, 15.5 and 5-25mm, and the 2 DCIS measured 38 and 58mm, respectively.

### Electronic format

Software was developed to display abMRI (RiViewer) [32] using a simplified display protocol (first post-contrast subtracted images (FAST MRI) displayed as maximum-intensity projection (MIP) and stacked, subtracted slices). Biopsy-proven cancers were drawn onto images electronically as ground truth. During hands-on workstation training (29 training abMRI scans), learners could discover the ground truth at the touch of a button, giving instant feedback (formative assessment).

The software contained an automatic timer to measure interpretation times.

The same software displayed the test set of 125 abMRIs [21]. The test set and the set of training scans were mutually exclusive. Training and test set MRIs were from a single centre but acquired during different years, from different women. The test set was presented to each reader in a different random order, and readers were unable to access the ground truth of the test set at any time (summative assessment).

### Standardised training

The structured training package [21, 27] was adapted to enable in person, small-group training, delivered by two radiologists experienced in fpMRI reporting Additional file 1 (Appendix 2: example study day agenda). Table 1 documents participants’ and trainers’ mammogram and fpMRI interpretation experience. Small-group presentations on aspects of abMRI interpretation alternated with guided hands-on workstation sessions to enable learners to practice image manipulation and abMRI interpretation on the training set of 29 abMRI scans. The presentations included multiple additional illustrative examples of abMRI images depicting specific learning points. These examples were taken from MRI scans not included in either the training or test sets.

**Table 1:** Demographics of participant mammogram readers and of the two trainers

	Group 1	Group 2	Trainers
Professional title*			
Advanced practitioner	0	12**	0
Consultant radiographer	0	5	0
Breast clinician	2**	4	0
Consultant radiologist	17**	1	2
*Professional experience*			
Number of years interpreting mammograms: median (range)	10 (1–25)	6 (<1–19)	13 (6–19)
Number of mammograms interpreted each year: median (range)	6000 (3000–13,000)	5,000 (4,500–11,600)	7500 (5000–10,000)
Participant readers who interpret digital breast tomosynthesis (DBT) in normal clinical practice	13	10	N/A
Number of years interpreting breast MRI: median (range)	6 (0.5–20)	N/A	10 (6–14)
Number of full-protocol breast MRI scans interpreted each year: median (range)	100 (40–350)	N/A	190 (180–200)
Total numbers of participant readers who attended the FAST MRI study day	19	22	N/A

The trainers were not study participants, and the details of their professional experience are provided for comparison only

*Professional titles in UK: Screening mammograms within the NHS Breast Screening Programme are interpreted by multidisciplinary healthcare professionals trained in mammogram interpretation. Their performance is subject to continuous audit through the UK Breast Screening Information System that produces individual real-life performance data over rolling 3-year periods (43)

“Consultant Radiologist” and “Breast Clinician” are titles held by medical doctors. Consultant Radiologists are registered on the General Medical Council’s Specialist Register following Completion of Specialist Training (5 years) with standards and curriculum set by the Royal College of Radiologists (RCR). The Association of Breast Clinicians launched the Credential in Breast Disease Management for Breast Clinicians, jointly with the RCR, in 2019, to standardise and formalise training for Breast Clinicians across the UK (3-year training programme)

“Advanced Practitioners” and “Consultant Radiographers” are experienced, registered healthcare practitioners, typically mammographers, who have additionally completed specialist training, underpinned by a master’s level award or equivalent to support their professional practice within the NHS (https://advanced-practice.hee.nhs.uk/)

**In total, 4 participant readers attended the training session but did not complete the follow-up dataset, namely one Consultant Radiologist, one Breast Clinician and two Advanced Practitioners

Throughout the training, mammogram readers’ prior knowledge was utilised and activated by repeated reference to similarities and differences between the two breast imaging modalities (abMRI and mammogram) and the varied appearances of cancer, and of other common breast pathologies, as displayed by each modality [33].

The training set was presented in batches, in the same order as in the previously reported one-to-one structured training package [21, 27], as guided hands-on workstation practice during which readers could discover the ground truth at the touch of a button, giving instant feedback to aid their learning (formative assessment).

Readers were taught how to classify abMRI scans according to the UK 5-point breast imaging classification specified for screening fpMRI in women at higher risk of breast cancer within NHSBSP [34, 35].

### Test set interpretation

Subsequent to completion of their training, readers interpreted the test set of 125 abMRIs [21], blinded to all other information (clinical history, previous imaging, histology and other readers’ interpretations). Readers were told to expect more cancers than in usual screening practice but no other indication of the number of cancers was given. The test set was presented to each reader in a different random order and readers were unable to access the ground truth at any time.

### Sample size calculation

Using the results of a previous single-centre study [21], a dataset of 250 breasts (125 women) allowed the lower 95% confidence limit of the inter-rater reliability to be estimated to within 0.07 with a minimum of 6 readers/group and a proportion of cancers of 0.22. Thus, we aimed for a minimum of 12 readers: 6 in each group.

### Statistical analysis

Per-breast analysis of the frequency of results against true outcome was obtained overall and for each reader. Sensitivity and specificity of readers’ abMRI classification with the true outcome were determined and differences across reader groups assessed using a multi-level-generalised-mixed model to account for multiple readers per scan and the dependence between breasts. The inter-reader variability and the agreement between readers and the true outcome were assessed using Cohen’s κ coefficient to account for the probability of agreement occurring by chance. Classifications 4 and 5 were considered indicative of cancer, and classifications 1–3 considered a normal result.

Interpretation times were compared across reader groups (Wilcoxon rank-sum). If readers returned to a scan on multiple occasions, interpretation times were calculated as total time spent on the scan.

To assess whether readers’ performance improved during the assessment task, the initial 55 scans interpreted by each reader (first set) and the subsequent 70 (second set) were compared overall and for each group.

A per-lesion analysis was also undertaken. Lesion localisation fraction (LLF) was calculated (number of true positives divided by total number of true cancers). For each reader group, a weighted jackknife alternative free-response receiver operator characteristics (JAFROC) curve was determined using the abMRI classifications for identified cancers, plotting the LLF against the false-positive fraction (fraction of normal breast with at least one false positive on its image). The empirical areas under the equally weighted JAFROC curve were used as figures of merit (FOM). Reader-averaged FOM for each group were compared using an analysis of variance (ANOVA) test. Data were analysed using SAS statistical software and “RJafroc” package within the R software.

Lastly, as a per-breast analysis, to simulate double reading (standard UK screening practice), results were calculated from randomly selecting two readers, and a third for arbitration of disagreement [36].

## Results

**Fig. 1:**
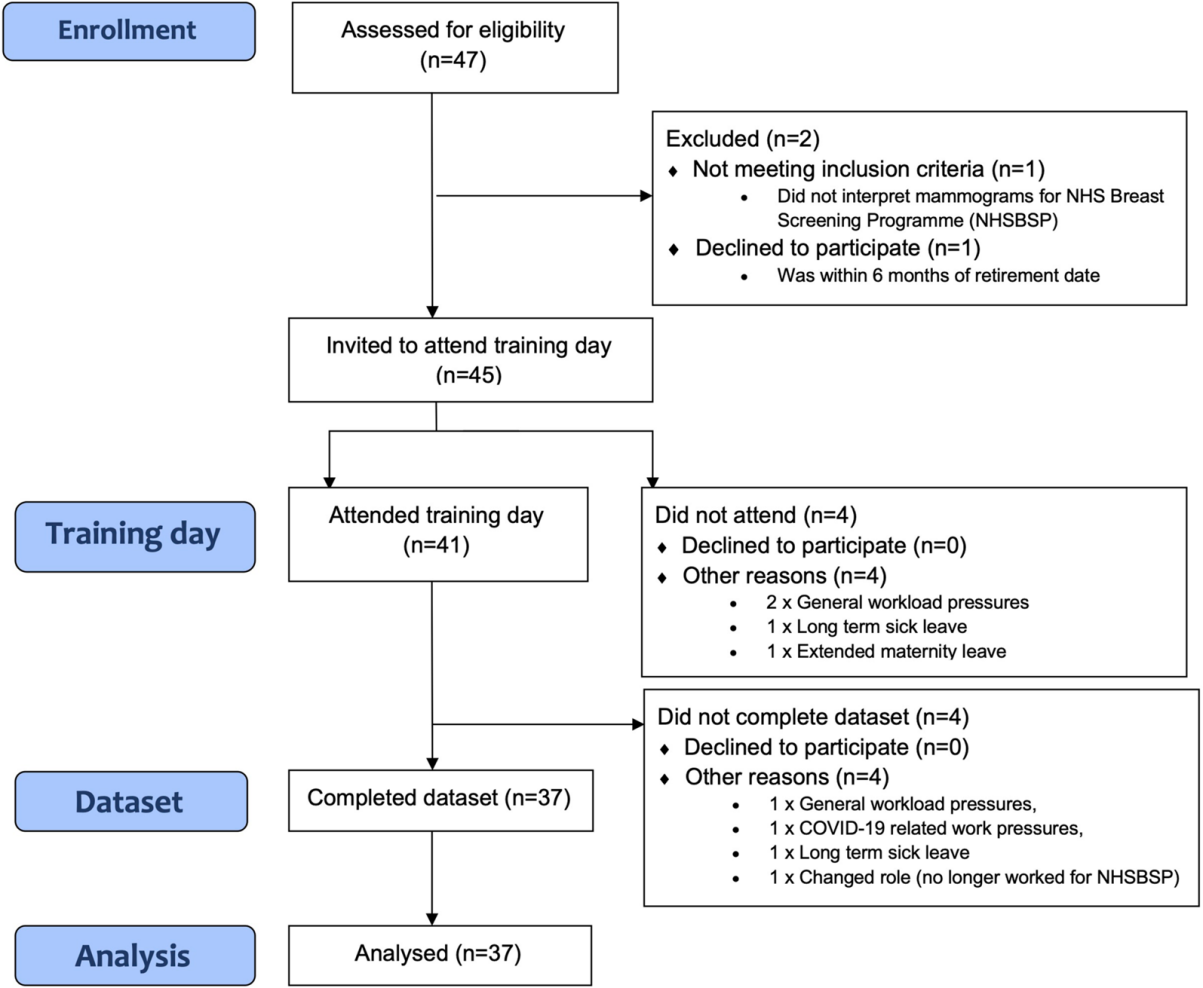
CONSORT flow diagram detailing participation in FAST MRI reader training programme

Thirty-seven participants (17 mammogram readers experienced in fpMRI interpretation (Group 1) and 20 mammogram readers without previous experience of fpMRI interpretation (Group 2)) completed both the training and the subsequent reading task, of the 125 abMRI test set (250 breasts), giving a total of 9250 reads. Figure 1 shows the flow chart of reader recruitment, and Table 1 details the professional roles and experience of the readers in each of the two groups. The training days were delivered by two authors (LJ and RG) whose professional roles and experience are also detailed in Table 1. Participant readers each attended a single training day and were trained in groups of 1-7 (median 4).

The readers were asked to review the training set prior to reading the test set, and they took a median time of 1 day for their review (interquartile range 0–9 days) with a maximum time of 30 days. The median time from attending the training day until starting the test set interpretations was 3.5 months (interquartile range 3.0–4.3 months) with a minimum time of 1.8 months and maximum of 8.9 months. The readers had access to the training set for further review at any time whilst reading the test set. During the training day, the participants had been given printed copies of the presentations and of other training materials and they were able to refer to these materials during their reading of the test set.

### Per-breast analysis

The per-breast analysis comparing readers’ MRI classification with the true outcome (cancer or normal) showed an overall sensitivity of 86% (95%CI 84–87%; 1776/2072) and specificity of 86% (95%CI 85–86%; 6140/7178).

**Fig. 2:**
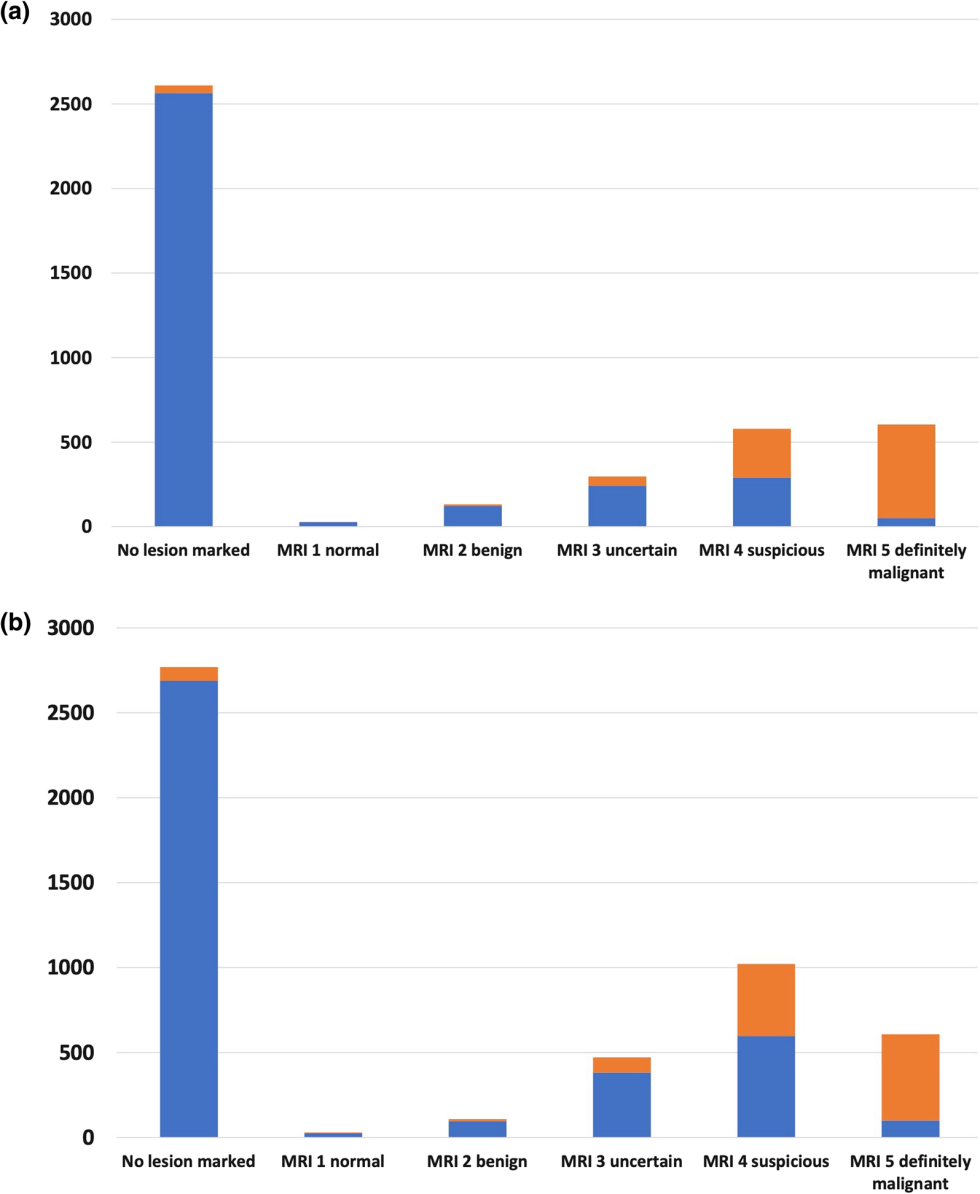
Bar chart showing the frequency of MRI classifications by whether there was a cancer or not present for each group of readers. **a** Group 1 **b** Group 2. Legend: Non-Cancer (Blue filled box), Cancer (Orange filled box)

Results for readers in Group 1 showed significantly higher sensitivity (843/952; 89%; 95%CI 86-91%) and higher specificity (2957/3298; 90%; 95%CI 89–91%) than Group 2 (sensitivity = 83%; 95%CI 81–85% (933/1120)* p* < 0.0001; specificity = 82%; 95%CI 81–83% (3183/3880) * p* < 0.0001). The Group 2 readers reported a higher proportion of MRI 4 classifications of 21% (1031/5000) compared to the 14% (579/4250) for the Group 1 readers, which led to the lower specificity achieved by Group 2 (Fig. 2). Inter-reader agreement was also higher for Group 1 (kappa = 0.73; 95%CI 0.68–0.79) than for Group 2 (kappa = 0.51; 95%CI 0.45–0.56) (Table 2).

**Table 2:** Comparison of readers’ MRI classification against true outcome (per breast)

	Ground truth result		Total	Kappa (95%CI)
	Cancer	Normal		
**Reader Classification**				
*All Readers*				
Cancer	1776	1038	2814	
No cancer	296	6140	6436	
Total	2072	7178	9250	0.63 (0.61–0.65)
*Group 1*				
Cancer	843	341	1184	
No cancer	109	2957	3066	
Total	952	3298	4250	0.72 (0.70–0.74)
*Group 2*				
Cancer	933	697	1630	
No cancer	187	3183	3370	
Total	1120	3880	5000	0.56 (0.54–0.59)

**Fig. 3:**
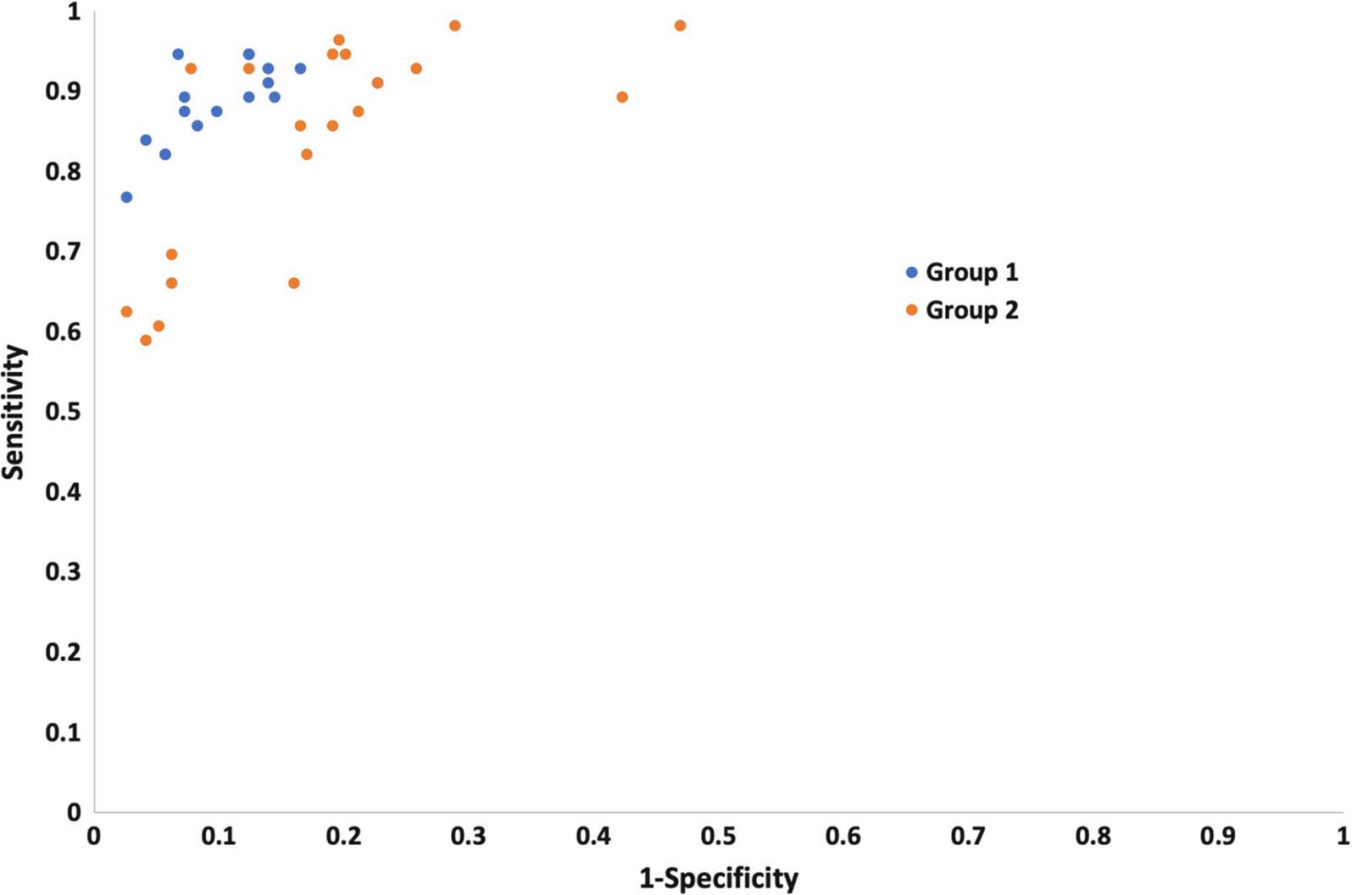
Point estimates of accuracy in receiver operating characteristic (ROC) space (sensitivity plotted against 1-specificity) for each reader in the study, coded by group (Group 1 = experienced breast MRI readers, Group 2 = mammogram readers who have undergone a single day’s training to interpret abbreviated breast MRI)

The receiver operating characteristics plot of the per-breast individual reader performance demonstrates that although the majority of Group 2 readers had a lower performance than the Group 1 readers, there were 5 Group 2 readers that showed similar levels of accuracy with the Group 1 readers (Fig. 3).

**Fig. 4:**
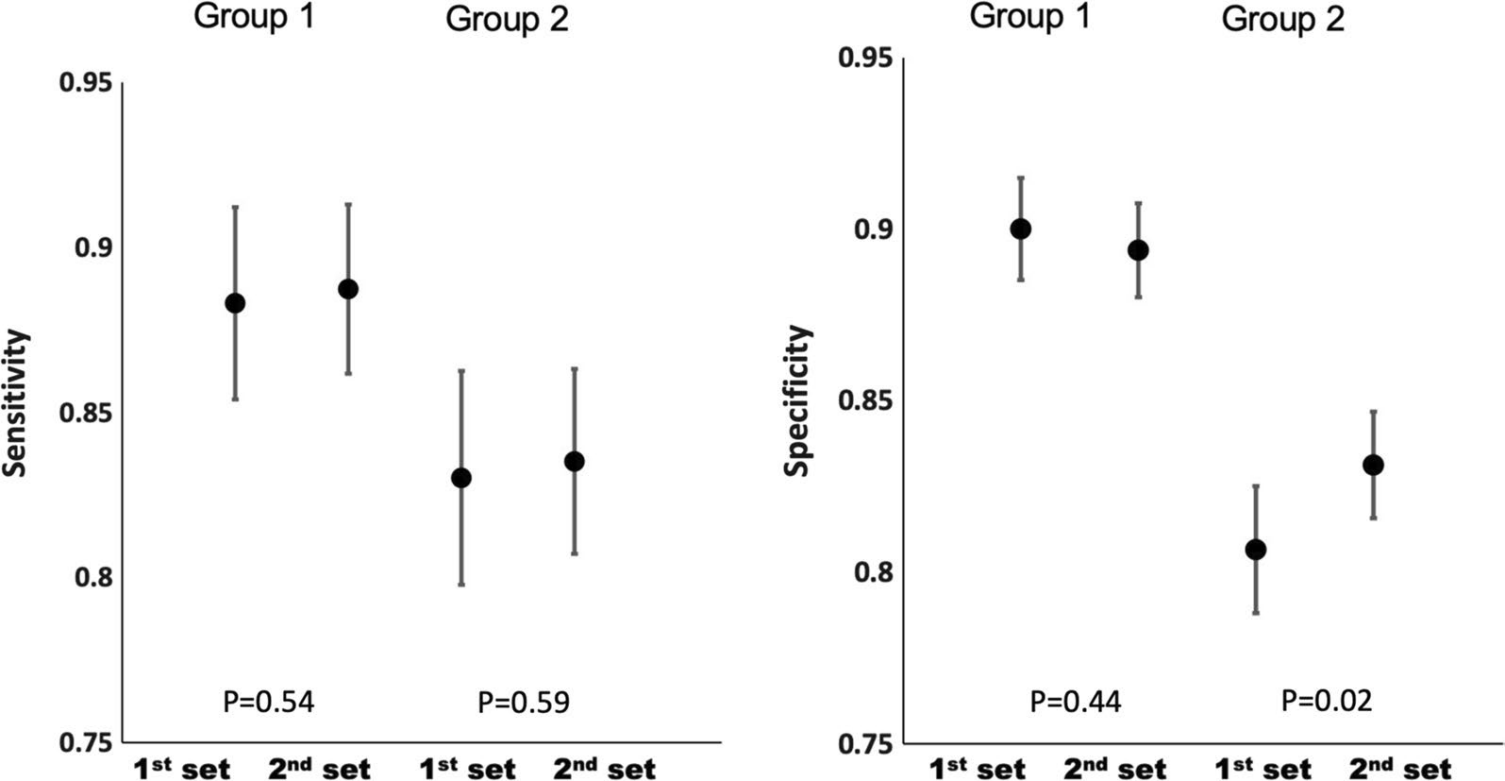
Sensitivity (95%CI) and specificity (95%CI) for the 1st set of 55 cases compared to 2nd set of 70 cases, demonstrating improvement in per-breast performance (specificity) for Group 2 but not for Group 1

There was a significant improvement in the specificity of the Group 2 readers from the first 55 scans interpreted to the remaining 70 scans from 81 to 83% (*p* = 0.02), whereas their sensitivity remained fairly consistent from 83 to 84% (*p* = 0.59). There were no significant improvements for the expert readers of Group 1, neither in sensitivity (from 88 to 89% (*p* = 0.54)) nor in specificity (from 90 to 89% (*p*= 0.44)) (Fig. 4).

### Time taken to report

**Fig. 5:**
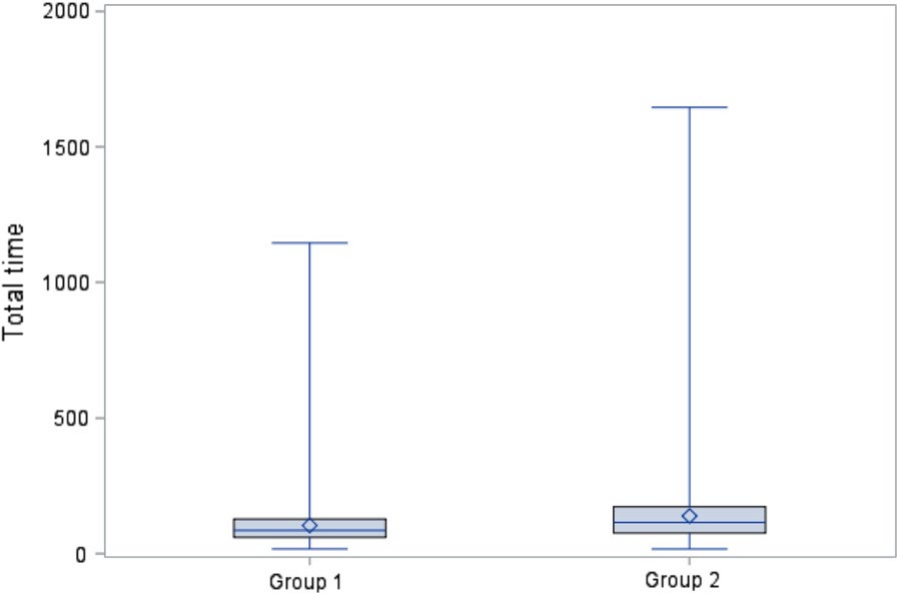
Box and whisker plot for total time taken (seconds) to report each case The long horizontal blue line represents the median; the top and the bottom of the box represent the 25th and 75th percentiles. The diamond in the box represents the mean. The vertical lines (*whiskers*) extend to the group minimum and maximum values. The outlier within Group 2 of 10003 seconds has been excluded for this plot. Wilcoxon rank-sum *p* < 0.0001.

The median time taken for individual readers to interpret each abMRI scan was 29 sec less for Group 1 (median 86 sec, range 17–1145 sec, interquartile range 60–127) than for Group 2 (115, 17-10003, 76–173 *p* < 0.0001) (Fig. 5). There were 25 (of 37) readers that returned to a total of 75 (of 125) scans on multiple times (range 1–15 scans) and there were 7 records (out of a total of 9250) where a reader took more than 1000 seconds to interpret. The interpretation time for both Group 1 and Group 2 readers decreased from the first 55 scans interpreted to the subsequent 70 (Group 1 median interpretation time decreased by 19.86 seconds (*p* < 0.0001), Group 2 by 31.11 (*p* < 0.0001)) (Table 3).

**Table 3:** Interpretation times compared across the sets of FAST MRI scans, overall and for each group of readers

Total time	Overall	Group 1	Group 2
*First set*			
Number	2035	935	1100
*Total time*			
Median	115.33	98.04	133.40
Interquartile range	74.25–173.49	65.56–142.26	84.89–196.05
Range	17.14–10003.38	17.14–1144.79	22.72–10003.38
*Second set*			
Number	2590	1190	1400
*Total time*			
Median	89.70	78.18	102.29
Interquartile range	62.26–136.11	55.23–114.16	71.62–150.54
Range	16.95–1645.50	117.99–796.06	16.95–1645.50
Wilcoxon rank-sum*	*p* < 0.0001	*p* < 0.0001	*p* < 0.0001

*The total interpretation times were compared across the sets, overall and for each group using a Wilcoxon rank-sum test.

### Per-lesion analysis

**Fig. 6:**
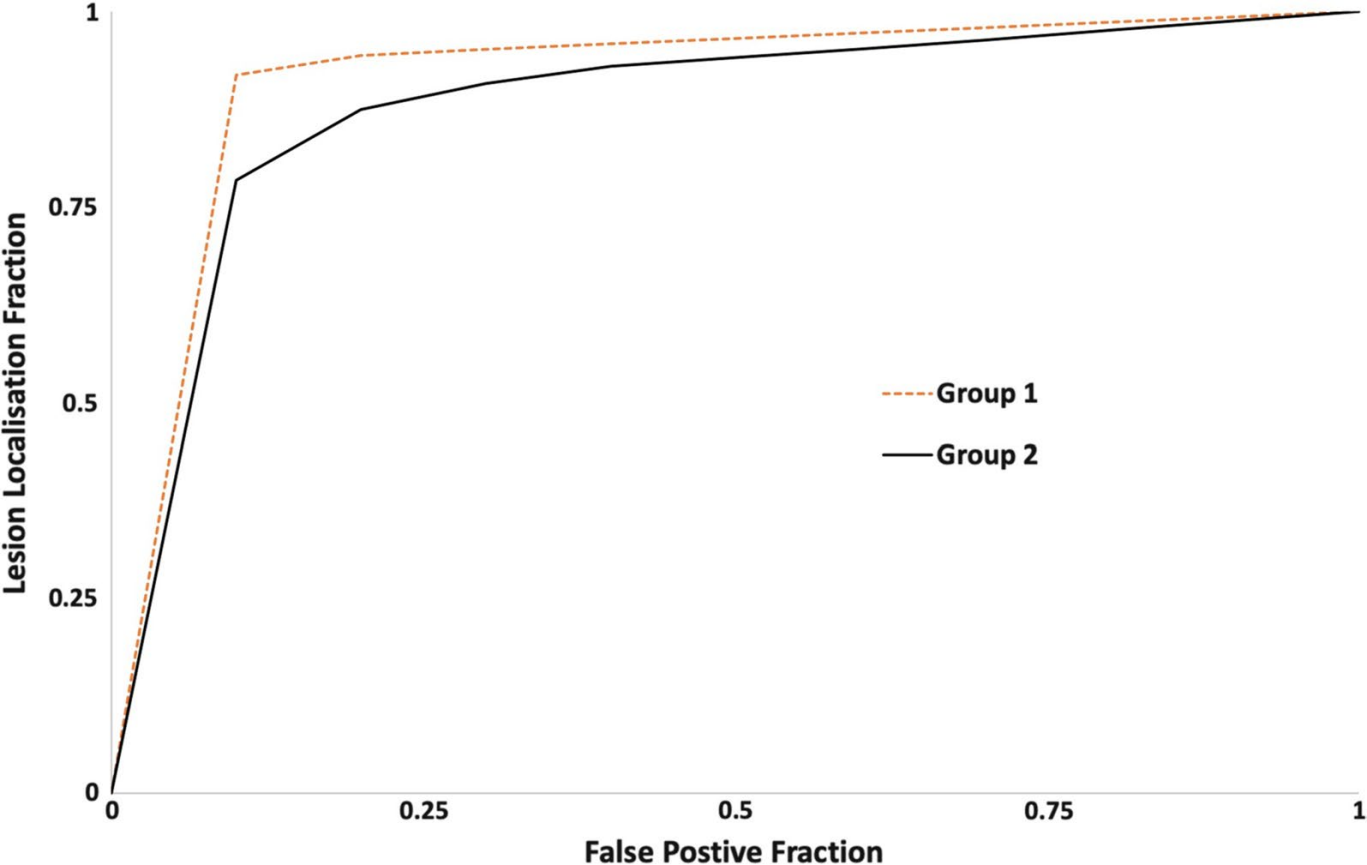
Per-lesion analysis demonstrated graphically as a jackknife alternative free-response receiver operator characteristics (JAFROC) curve There were 58 biopsy-confirmed cancer lesions in the dataset, equating to a total of 2146 decisions made by the 37 readers. The LLF was overall was 83% (1783/2146); 86% (847/986) for the Group 1 readers and 81% (936/1160) for the Group 2 readers. The reader-averaged weighted JAFROC FOM was 0.93 (95%CI 0.92–0.94) overall. The reader-averaged weighted JAFROC FOM for Group 1 readers of 0.95 (95%CI 0.95–0.96) was significantly higher than for Group 2 (0.91; 95%CI 0.89–0.93); *p* = 0.004

There were 58 biopsy-confirmed cancer lesions in the dataset, equating to a total of 2146 decisions made by the 37 readers. The LLF was 83% (1783/2146) overall, 86% (847/986) for the Group 1 readers and 81% (936/1160) for the Group 2 readers. The reader-averaged weighted JAFROC FOM was 0.93 (95%CI 0.92–0.94) overall. The FOM for Group 1 readers of 0.95 (95%CI 0.95–0.96) was significantly higher than for Group 2 (0.91; 95%CI 0.89–0.93); *p* = 0.004) (Fig. 6).

### Per-woman analysis

**Fig. 7:**
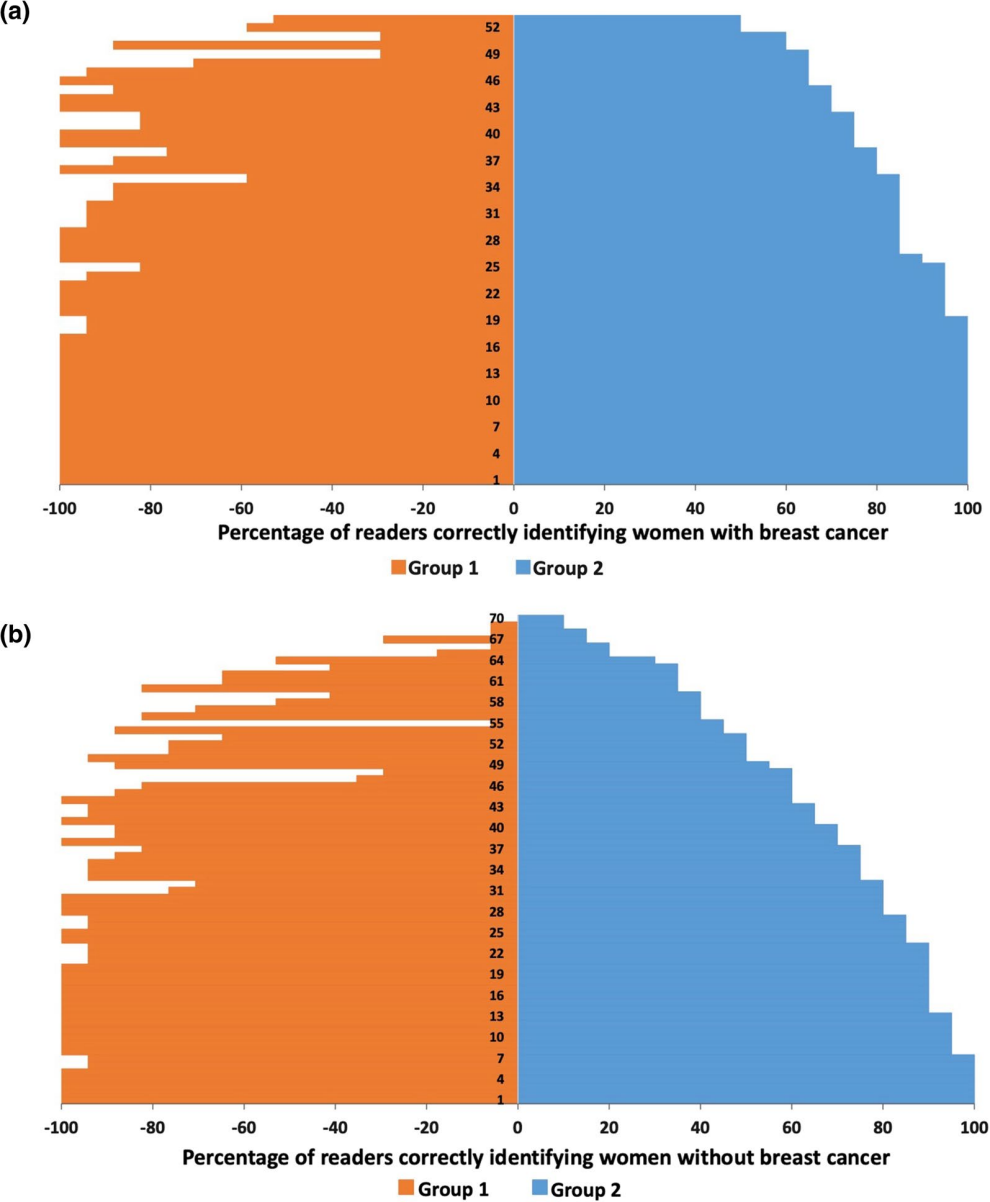
Per-woman abbreviated breast MRI (abMRI) analysis** a** Illustration of the percentage of readers correctly identifying each of the 55 women with breast cancer** b** Illustration of the percentage of readers correctly identifying each of the 70 women without breast cancer

On a per-woman basis, the 125 women (whose abMRI scans comprised the test set) were reported by the 37 readers, giving a total of 4625 reads. Thirty-eight percent of the women (1744/4625) were correctly identified as having cancer and therefore would have been correctly recalled if their abMRIs had been single read, whilst 41% women (1898/4625) were correctly identified as not having cancer and would not have been recalled. A further 10/4625 (< 1%) women with cancer would have been recalled, but incorrectly, based on a different lesion. In total, 15% women (692/4625) would have been incorrectly recalled but found not to have cancer and 6% women (281/4625) with cancer would have been missed and not recalled if single read (Fig. 7). These figures represent single reading of the test set by all readers (Group 1 and Group 2) and equate to a per-woman sensitivity of 86% (1744/2035) and specificity of 73% (1898/2590), with 14% false negatives (281/2035) and 27% false positives (692/2590).

### Double-reading simulation analysis for the consecutive series of screening cases

The enriched test set of 125 abMRI scans included 72 consecutive screening cases, and the results for these 72 women alone (consecutive screening subset) were re-analysed on a per-breast basis to simulate double reading (standard practice in NHSBSP). Using two random readers and a third for arbitration when there was disagreement, there were 144 breast results comprising two breasts with biopsy-proven cancer and 142 without cancer (at least 2-year normal follow-up).

There were 124/144 (86%) breasts correctly identified as not having cancer, and both breasts with cancer were correctly identified as having cancer (2/144 (1%)). There were no false negatives. However, 18/144 (13%) breasts were incorrectly identified as having cancer. Hence, sensitivity was 100% (85% CI 16–100%) with 87% (95%CI 81–92%) specificity.

Of the 18 false-positive breasts, the original fpMRI reports (unavailable to readers) contained the following information (unavailable to the blinded readers in this study): 6 lesions were noted on the original 2015 fpMRI report to be unchanged since a previous fpMRI, 3 had previously been biopsied and were therefore known to be benign at the time of reporting in 2015, 1 had a recent biopsy noted in the 2015 report that explained the positive finding and 2 had been recalled in 2015 from the fpMRI for the same finding which was subsequently demonstrated as benign by either biopsy or follow-up.

## Discussion

### Summary of findings

Following a single day of standardised, small-group training, mammogram readers’ overall diagnostic performance at abMRI interpretation achieved benchmarks set for fpMRI by the American College of Radiology’s Breast Imaging Reporting and Data System (BI-RADS) for both sensitivity (86% achieved vs. >80% BI-RADS benchmark [37]) and specificity (86% achieved vs. >85% BI-RADS benchmark [37]).

The performance of readers experienced in interpreting fpMRI (Group 1) was significantly better than that of mammogram readers without previous experience in fpMRI interpretation (Group 2): sensitivity (*p* < 0.0001), specificity (*p* < 0.0001) and inter-reader variability (non-overlapping 95%CIs).

The performance of the novice readers of Group 2 improved during the reading task (*p* = 0.02), whereas that of the expert readers of Group 1 did not. This improvement in performance of Group 2 readers occurred despite them receiving no feedback about the ground truth of scans during the reading task.

### Literature comparison with reader results in this study—diagnostic accuracy

A European multi-reader study of ultrafast breast MRI interpretation of an enriched dataset by 7 breast radiologists, with 6–15 years’ experience in fpMRI interpretation, compared diagnostic performances at ultrafast breast MRI and fpMRI. Their readers’ diagnostic performance at ultrafast (sensitivity: 84% and specificity 82%) was similar to that of our novice Group 2 readers, who had no previous experience in breast MRI interpretation (sensitivity: 83% and specificity: 82%). Their inter-reader agreement (kappa=0.73) was similar to that of our fpMRI-experienced, Group 1 readers (0.71), whilst our FOM from the per-lesion JAFROC analysis (Group 1: 0.95, Group 2: 0.91 and overall: 0.93) compared well with their non-localised AUC (0.89) [38].

Single-reading diagnostic performance at abMRI of the novice Group 2 readers in the current study was similar to published figures for diagnostic performance at fpMRI for radiologists, experienced in breast MRI interpretation, in community screening practice in the USA (13,000 fpMRI examinations reported by the Breast Cancer Surveillance Consortium (BCSC): sensitivity: 83% Group 2 vs. 81% BCSC and specificity: 82% Group 2 vs. 83% BCSC) [39].

The published EA1141 trial reported diagnostic accuracy for abMRI, single read (standard USA practice) by experienced fpMRI readers who had successfully completed the Society of Breast MRI’s abMRI interpretation course. The diagnostic accuracy its abMRI readers achieved (sensitivity: 95.7% and specificity: 86.7%) [1] is similar to the results of our double-reading simulation analysis of the consecutive screening subset of scans within our test set (Groups 1 and 2 readers combined) of 100% sensitivity and 87.3% specificity. Double reading is standard UK practice.

In a study of 116 Australian breast radiologists, interpreting screening mammograms for population risk women under test conditions, outside clinical practice [40], their overall JAFROC score was 0.78 (95%CI 0.77–0.80), whilst that of the subset of radiologists who read >5000 mammograms/year was higher: 0.86 (95%CI 0.83–0.88). Both these figures are lower than the equivalent figures (FOM) obtained for FAST MRI by our readers (Group 1: 0.95, Group 2: 0.91 and overall: 0.93). In addition, our readers’ figures for LLF (overall: 0.83; Group 1: 0.86 and Group 2: 0.81) are also considerably higher than the equivalent figures for location sensitivity achieved for mammography by the Australian radiologists (overall: 0.56, and for the subset of radiologists reading > 5000 mammograms/year: 0.59). These differences in LLF highlight the greater inherent sensitivity in the technique of FAST MRI in comparison with mammography, as FAST MRI was designed to expand the indications for breast MRI into populations of women currently screened with mammography, such as women with mammographically dense breasts who are otherwise at population risk of breast cancer but whose cancers are often missed by mammography [1, 3].

### Literature comparison with reader results in this study—reading times

The median reading times, of both the expert readers (Group 1) and novice readers (Group 2) in the current study, fall within the range of times to interpret abMRI reported in the literature [11, 13, 14, 16, 18], despite the automated recorded timings including time taken by readers to electronically complete required answers for each case about background parenchymal enhancement and motion artefact. Group 2, on average, took approximately a third longer than Group 1 to interpret abMRI (median 115 sec vs. 86). Whilst time taken to interpret abMRI significantly shortened during the reading task for both Groups 1 and 2, allowance for longer interpretation times by Group 2 readers should be factored into workforce planning around this new technology.

### Study limitations

In this study, readers only interpreted the test set on one occasion and so we can provide no information on intra-reader variability.

The study used an enriched test set that included cancers from symptomatic breast practice which are not representative of MRI screening detected cancers. This was discussed in a previous publication of this dataset [21]. Limitations of this study also include that the test set was not read within clinical practice, exposing the readers to a negative “laboratory effect” on their performance [41].

In the current study, we included an additional analysis of the small, consecutive screening series [31] (72 abMRI scans, a subset of the enriched test set), without enrichment. Simulated double-reading, per-breast analysis of this consecutive screening series subset of scans was performed using the relatively large number of independent blinded reader interpretations obtained. However, although we used the proxy for arbitration that was available to us, we understand that arbitration in practice would be different and could therefore yield different results.

### Double-reading simulation

The double-reading simulation enabled a tentative prediction of the potential diagnostic accuracy that using abMRI, double-read by trained mammogram readers (those with previous experience of fpMRI interpretation and those without) might achieve if used to screen this population.

Double-reading simulation analysis of the consecutive screening subset suggested correct identification of both of the 2 breasts with cancer for a recall rate of 20/144 (14%). In our study, double reading of abMRI was entirely blinded to past history and previous imaging, unlike in clinical practice. Of the 18/144 false-positive assessments made by double-reading simulation in this study, interrogation of the original 2015 fpMRI reports, revealed past history information and/or previous imaging information (unavailable to our readers during the study) that would have obviated the need for recall in 10 of the 18 false-positive double reads, potentially reducing the recall rate to 6% (8/144) with no detriment to sensitivity (100%; 2/2).

There is evidence that in the practice of screening mammography, reader diagnostic performance is related to the annual number of mammograms read and the number of years of experience in mammogram interpretation [40]. It may be that the diagnostic performance achieved by our novice Group 2 readers from a single day of training could be sufficient for them to join their fpMRI-experienced Group 1 colleagues in contributing to double reading of abMRI, provided there were initial individual credentialing by sensitivity and specificity threshold and ongoing audit and performance assessment, similar to that currently in place for mammogram readers within NHSBSP (Breast Screening Information System (BSIS) [42] and Personal Performance in Mammographic Screening (PERFORMS) [43]). These systems could chart the improvement likely to occur in mammogram readers’ performance with increasing abMRI interpretation experience.

### Implications of the research

**Fig. 8:**
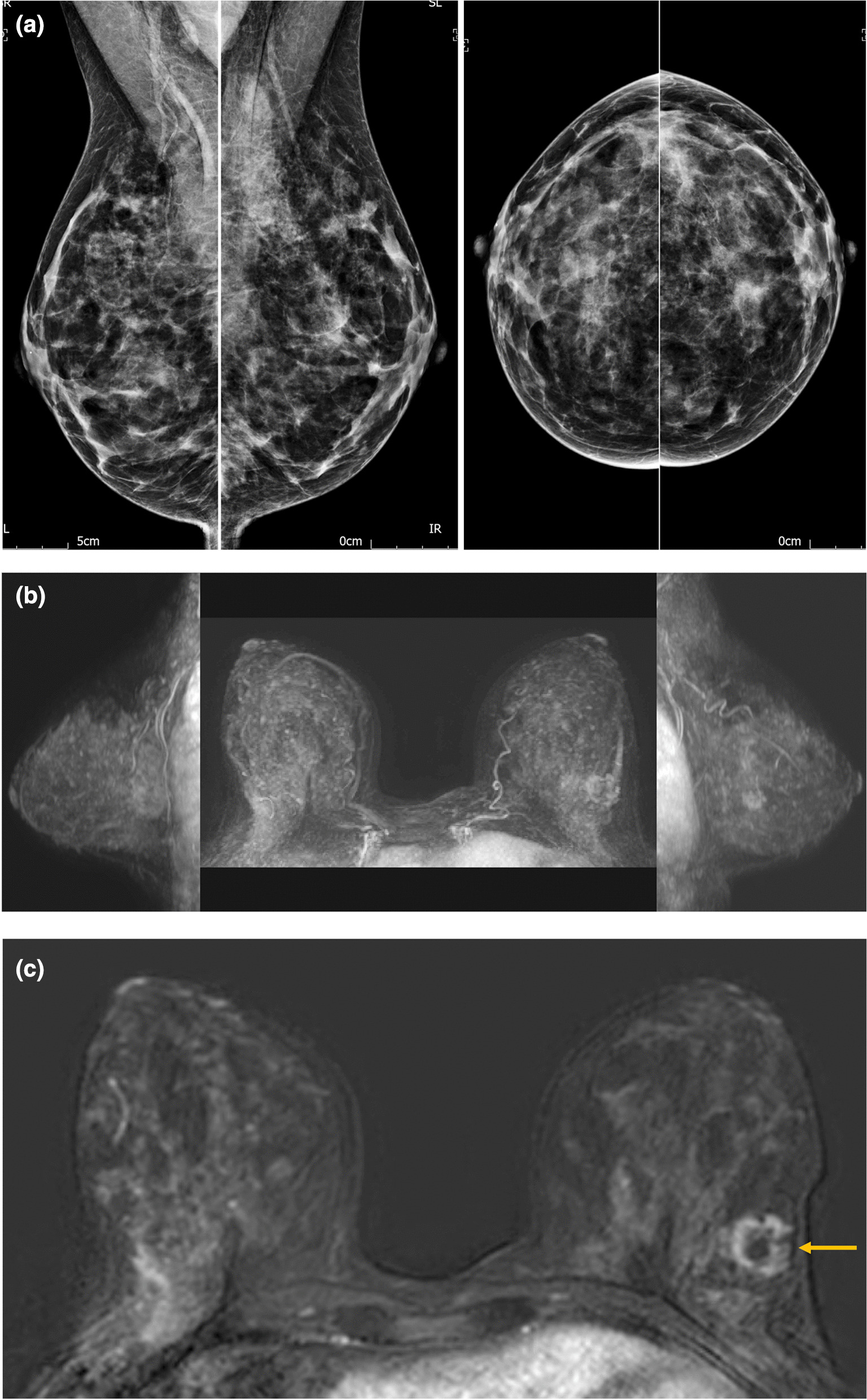
**a**–** c**: Example cancer case from the dataset. This 25mm diameter Grade 2 carcinoma of no special type (oestrogen receptor positive, progesterone receptor equivocal, Her2 receptor negative and Ki67: 20%) was occult mammographically (**a**) because it was obscured by mammographically dense fibroglandular tissue. It was seen only subtly on the maximum intensity projection (MIP) image of the FAST MRI (**b**) as it was partially obscured by background parenchymal enhancement (BPE). However, it is clearly seen as a rim enhancing mass on the FAST MRI stack of slices (**c**), indicated by a yellow arrow. It was correctly identified as a cancer by 17/17 Group 1 readers and 17/20 Group 2 readers during this study.

Figure 8 illustrates an example cancer case from the test set. The 25 mm Grade 2 carcinoma of no special type was occult mammographically and is demonstrated only subtly on the FAST MRI MIP image but clearly visible on images from the FAST MRI stack of slices. It was correctly identified as a cancer by 17/17 Group 1 readers and 17/20 Group 2 readers during the study. The enriched test set used in this study (Appendix 1) was developed to be a challenging test of performance for novice abMRI readers [21] and included 56 breasts with cancer, of which 25/56 had lobular histology, more difficult to detect at mammography than cancers with other histology [44] and an additional 18/56 breasts with cancers that were mammographically occult in clinical practice (including the case illustrated in Fig. 8).

The improvement in performance demonstrated during the reading task by our novice readers indicates the presence of a learning curve for this group, and the results of this study suggest it is likely that additional training will enhance the performance of these readers in terms of both diagnostic accuracy and interpretation speed. Further research is needed, to explore additional training of mammogram readers new to breast MRI interpretation, to map their learning curve.

The results of this study showed, as expected, that a single day of abMRI interpretation training is insufficient for the diagnostic accuracy of mammogram readers new to breast MRI interpretation (Group 2) to match that of those experienced in fpMRI interpretation (Group 1). However, overall diagnostic accuracy for single reading by the two groups of readers combined was within published benchmarks, and the single day of training enabled the multi-professional mammogram readers new to breast MRI interpretation (Group 2) to achieve diagnostic performance comparable with that published for radiologists experienced in breast MRI in community screening practice [39].

The similarity of the results achieved by the double-reading simulation of the consecutive screening series subset of the test set to those achieved in the published EA1141 trial of abMRI (in which scans were single reported by radiologists experienced in fpMRI following additional abMRI interpretation training) suggests that the current study’s standardised single-day training may be sufficient for mammogram readers to commence their contribution to double reading of abMRI with appropriate initial credentialing and ongoing audit of performance.

Given that breast MRI is much better at detecting high grade, aggressive cancers at a smaller size than mammography [2, 3, 45] and early detection of breast cancer improves survival [46, 47], upscaling abMRI provision and augmentation of the current fpMRI interpretation workforce through the development of standardised, effective training and performance evaluation is a priority for the specialty. Prospective feasibility research to investigate current uncertainties around recall rates and rates of image-guided core biopsy, vacuum-assisted biopsy and MRI-guided biopsy will be another necessary research step to enable cost-effectiveness analysis of the use of abMRI as a screening tool and will inform decisions by policy makers about the potential introduction of abMRI into future clinical screening practice.

## Conclusions

Single-day abMRI interpretation training achieved diagnostic performance, at single read, for NHSBSP mammogram readers within benchmarks published for fpMRI.

The single day of training was insufficient for diagnostic accuracy of mammogram readers new to breast MRI to match that of experienced fpMRI readers but may be sufficient for their contribution to double reading.

Performance of novice abMRI readers showed in-task improvement, indicating a learning curve (potential for improvement with additional training).

## Supplementary Information

**Additional file 1**. **Appendix 1:** Specification of the abbreviated breast MRI (abMRI) protocol and composition of the FAST MRI test set used in the study. **Appendix 2:** Example of FAST MRI Study Day Agenda (identifiable information redacted for inclusion inblinded manuscript).

## Data Availability

The dataset generated and analysed during the current study is not yet publicly available because it is currently being developed into a publicly shareable format. Instead, it is available from the corresponding author on reasonable request.

## Abbreviations

abMRI: Abbreviated breast magnetic resonance imaging
ACR: American College of Radiology
ANOVA: Analysis of variance
AUC: Area under a curve
BCSC: Breast Cancer Screening Consortium
BSIS: Breast Screening Information Service
BI-RADS: American College of Radiology’s Breast Imaging Reporting and Data System
CI: Confidence interval
DCIS: Ductal carcinoma in situ
EUSOBI: European Society of Breast Imaging
FAST MRI: First post-contrast subtracted images (breast MRI)
FOM: Figures of merit
fpMRI: Full-protocol breast magnetic resonance imaging
IRAS: Integrated Research Application System for applications to the Health Research Authority and the Research Ethics Committee
ISRCTN: International Standard Randomised Controlled Trial Number (however, over the years the scope of the registry has widened beyond randomised controlled trials to include any study designed to assess the efficacy of health interventions in a human population.)
JAFROC: Jackknife alternative free-response receiver operator characteristics
LLF: Lesion localisation fraction
MIP: Maximum-intensity projection image
MRI: Magnetic resonance imaging
NHSBSP: National Health Service (United Kingdom) Breast Screening Programme
PERFORMS: Personal Performance in Mammographic Screening
REC: Research Ethics Committee
UK: United Kingdom of Great Britain and Northern Ireland
USA: United States of America
