# Supplementary material for: Evaluating the effectiveness of abbreviated breast MRI (abMRI) interpretation training for mammogram readers: a multi-centre study assessing diagnostic performance, using an enriched dataset
Source: Breast Cancer Res. 2022 Jul 30;24:55. doi: 10.1186/s13058-022-01549-5 (PMC9338668; doi:10.1186/s13058-022-01549-5)
Supplement: Supplementary file 1 — Additional file 1. Appendix 1: Specification of the abbreviated breast MRI (abMRI) protocol and composition of the FAST MRI test set used in the study. Appendix 2: Example of FAST MRI Study Day Agenda (identifiable information redacted for inclusion inblinded manuscript). [file 13058_2022_1549_MOESM1_ESM.docx]

# Additional File 1

## Appendix 1: Specification of the abbreviated breast MRI (abMRI) protocol and composition of the FAST MRI test set used in the study

The following text has been reproduced from: ﻿Jones LI, Geach R, Harding SA, Foy C, Taylor V, Marshall A, et al. “Can mammogram readers swiftly and effectively learn to interpret first post-contrast acquisition subtracted (FAST) MRI, a type of abbreviated breast MRI?: a single centre data-interpretation study”. **Br J Radiol 2019**; 92: 20190663 (18). The British Institute of Radiology (BIR) holds the copyright for the original article. The following reproduction of the text is in line with BIR policy: <https://www.birpublications.org/page/permissions#:~:text=the%20original%20source.-,Are%20you%20the%20author%20of%20the%20original%20article%3F,free%20of%20charge%20providing%20they%20cite%20the%20original%20source%20article.,-BJR%7Ccase%20reports> (last accessed 19/04/2022).

### Breast MRI protocol

All MRI examinations in the dataset were originally acquired on either a Philips (Amsterdam, Netherlands) Ingenia 1.5T or a Philips Ingenia 3T scanner. The breast coils used were dStream Breast seven-channel coils. The paramagnetic contrast agent used was gadobutrol 1.0 mmol ml−1 and the dose administered was 0.1 ml gadobutrol per kg body weight. The dynamic sequence used (from which the dataset’s FAST MRI images were obtained through post-processing) was dyn_eTHRIVE (Axial 3D T1 fast field echo (FFE), TR/TE 5.1/2.8 with 10 degree flip angle and SPAIR Power two fat suppression). Post-contrast scan commenced contemporaneous with the commencement of contrast injection (average duration 1.08 minutes)). Since the images used in the current study were originally acquired in 2015 and then later reprocessed and anonymised for the study, the acquisition protocol conformed to our own centre’s standard. This differed from Kuhl’s description of FAST MRI (11) as follows:

1. The breasts were not compressed during MR acquisition.
2. (ii) The T1 images of the dynamic study that were used to form the subtracted images were fat-suppressed (dyn_eTHRIVE).

The MRI scans performed for a screening indication were performed during day 6–16 of the woman’s menstrual cycle, but those performed post cancer diagnosis were performed promptly without reference to the woman’s menstrual cycle.

The MRI studies were copied, anonymised and allocated study identifiers chronologically for the date they were acquired and, as a consequence, normal and abnormal scans were presented to the readers in an unpredictable order. They were then reduced to comprise simply those MR sequences that would have been obtained if they had originally been acquired as a FAST MRI, displayed as an axial maximum intensity projection image (MIP), and also as a stack of axial slices (slice stack) of the first post-contrast-subtracted images from the dynamic series of the breast MRI examination. This process was performed by two of the research team who were not subsequently part of either of the two reading groups. These subtracted images alone comprised the FAST MRI scans interpreted by the readers.

##

### Composition of the FAST MRI test set used in the study

The following figure and table have been reproduced from: ﻿Jones LI, Geach R, Harding SA, Foy C, Taylor V, Marshall A, et al. “Can mammogram readers swiftly and effectively learn to interpret first post-contrast acquisition subtracted (FAST) MRI, a type of abbreviated breast MRI?: a single centre data-interpretation study”. **Br J Radiol 2019**; 92: 20190663 (18). The British Institute of Radiology (BIR) holds the copyright for the original article. The following reproduction of the text is in line with BIR policy: <https://www.birpublications.org/page/permissions#:~:text=the%20original%20source.-,Are%20you%20the%20author%20of%20the%20original%20article%3F,free%20of%20charge%20providing%20they%20cite%20the%20original%20source%20article.,-BJR%7Ccase%20reports> (last accessed 19/04/2022).


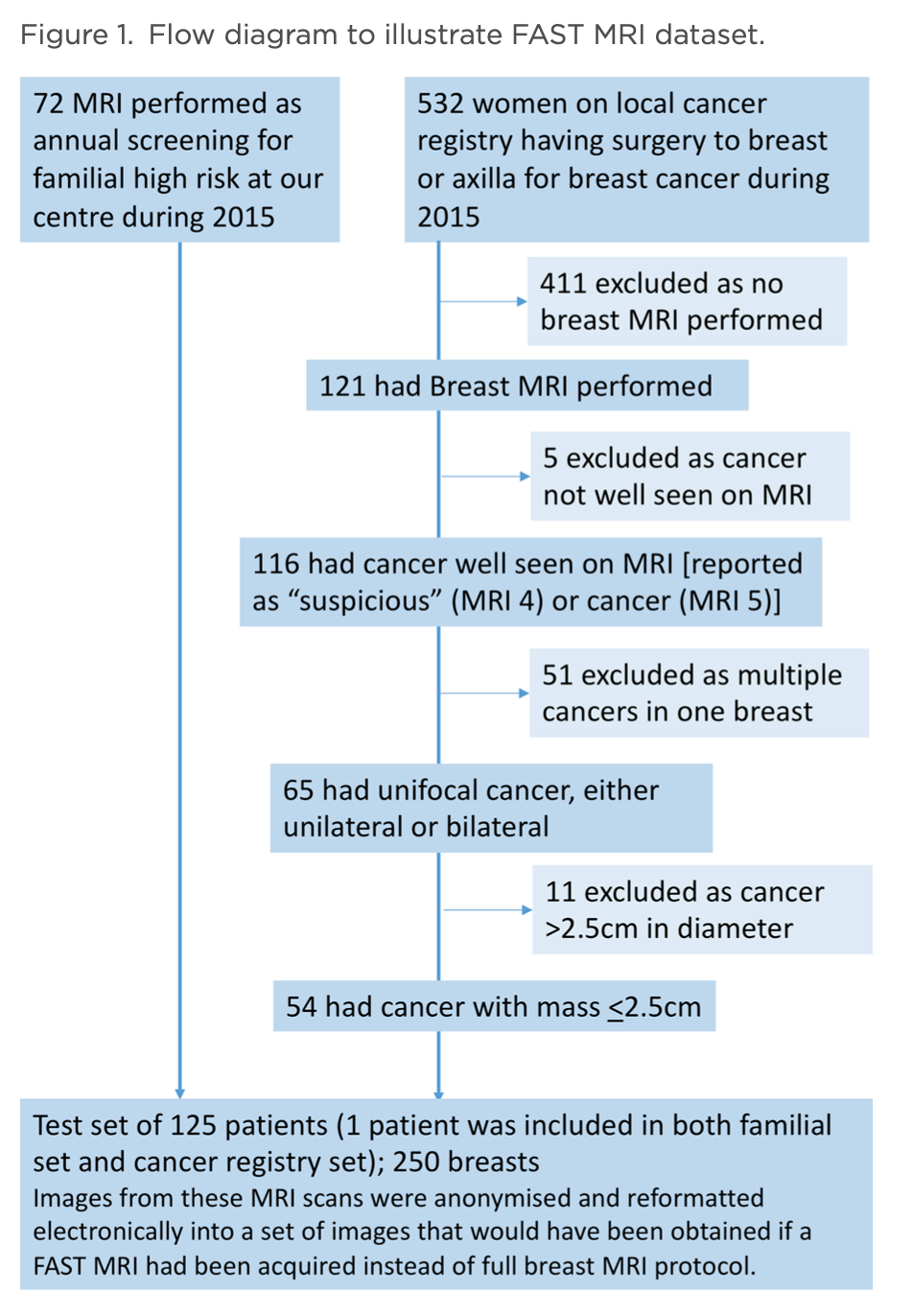


##
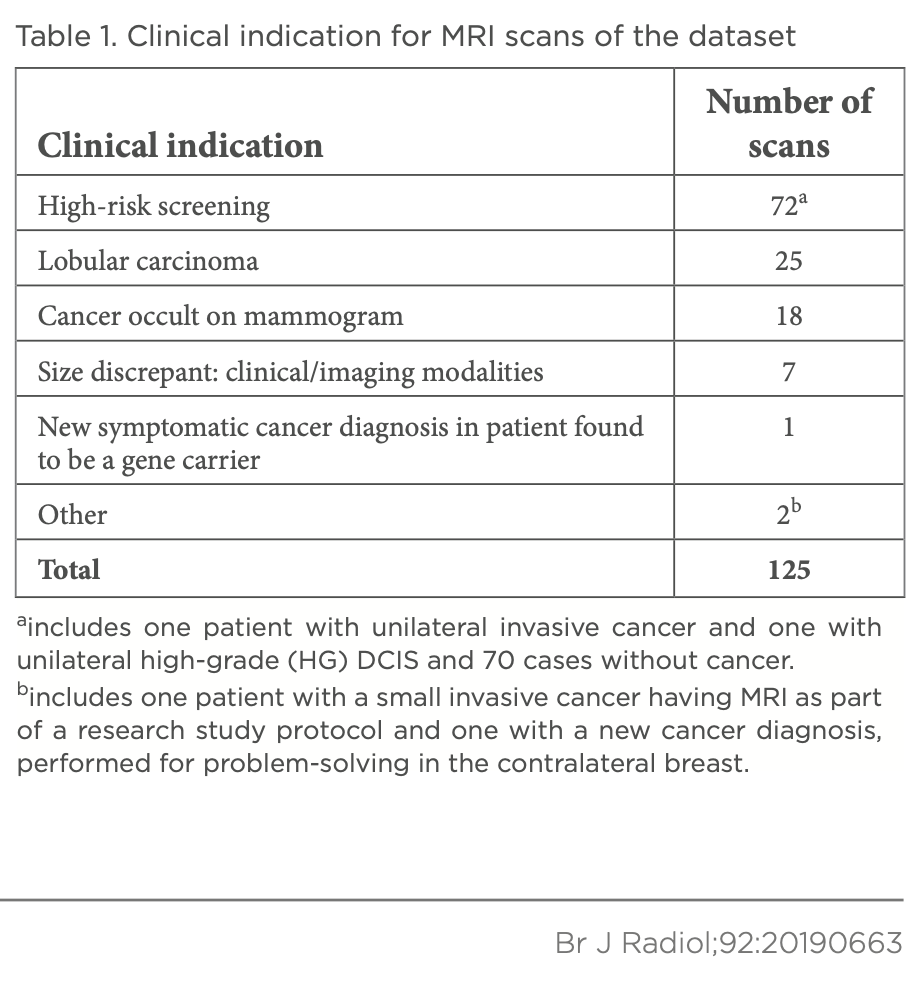


## Appendix 2: Example of FAST MRI Study Day Agenda (identifiable information redacted for inclusion in blinded manuscript)
